# Structure, Shift in Redox Potential and Li-Ion Diffusion Behavior in Tavorite LiFe_1−*x*_V*_x_*PO_4_F Solid-Solution Cathodes

**DOI:** 10.3390/molecules24101893

**Published:** 2019-05-16

**Authors:** Jia-Li Yan, Gang-Qin Shao, Shu-Hao Fan, Can Zhu, Yong Zhang, Jun Wang, Qi Liu

**Affiliations:** State Key Laboratory of Advanced Technology for Materials Synthesis and Processing, Wuhan University of Technology, Wuhan 430070, China; 211689@whut.edu.cn (J.-L.Y.); shuhao_fan@163.com (S.-H.F.); zhucan@whut.edu.cn (C.Z.); 223117@whut.edu.cn (Y.Z.); wangjun9413@whut.edu.cn (J.W.); 593859987@whut.edu.cn (Q.L.)

**Keywords:** LiFePO_4_F–LiVPO_4_F, solid solution, single-phase reaction, redox potential, diffusion coefficient

## Abstract

Solid-solution Li-ion cathode materials transform through a single-phase reaction thus leading to a long-term structural stability and improved cyclability. In this work, a two- to single-phase Li^+^-extraction/insertion mechanism is studied through tuning the stoichiometry of transition-metal Fe/V cations to trigger a transition in the chemical reactivity path. Tavorite triclinic-structured LiFe_1−*x*_V*_x_*PO_4_F (*x* = 0, 0.1, 0.3, 0.5, 0.7, 0.9, 1) solid-solution powders were prepared by a facile one-step solid-state method from hydrothermal-synthesized and commercial raw materials. The broad shape of cyclic voltammetry (CV) peaks, sloping charge/discharge profiles and sloping open-circuit voltage (OCV) profiles were observed in LiFe_1−*x*_V*_x_*PO_4_F solid-solution cathodes while 0 < *x* < 1. These confirm strongly a single-phase behavior which is different from the two-phase behavior in the end-members (*x* = 0 or 1). The electronegativity of *M* (*M* = Fe_1−*x*_V*_x_*) for the redox potential of Fe^2+/3+^ couple or the *M*–O_4_F_2_ bond length for the V^3+/4+^ couple plays respectively a dominant role in LiFe_1−*x*_V*_x_*PO_4_F solid-solution cathodes.

## 1. Introduction

Tavorite-structured (*P*$\bar{1}$, triclinic) lithium transition-metal fluorophosphates Li*M*PO_4_F (*M* = Fe, V) with 3D Li^+^-diffusion channels have been proposed as alternative cathode candidates for Li-ion batteries after the olivine-structured LiFePO_4_ (with 1D channels) was invented. The ionic conductivity of LiFePO_4_F (0.6 × 10^−7^ S cm^−1^ [1]) is about two orders of magnitude higher than that of LiFePO_4_ (~1 × 10^−9^ S cm^−1^ [2]). But the potential of Fe^2+/3+^ redox couple in the former is lower than the latter (~2.8 [3,4] vs. ~3.5 V [5]). This can be tuned through the inductive effect introduced by the V^3+/4+^ couple (~4.28 V [6,7]) to form LiFe_1−*x*_V*_x_*PO_4_F (0 ≤ *x* ≤ 1) solid solutions. Here we noticed that the specific capacity of LiVPO_4_F is almost the same as that of LiFePO_4_F (151.6 vs. 155.9 mAh g^−1^), and the specific energy of LiVPO_4_F [8,9,10,11,12,13,14,15,16,17,18,19,20] is larger than that of LiFePO_4_F [4,21,22,23] (667 vs. 424 Wh kg^−1^).

Solid-solution Li-ion cathode materials transform through a single-phase reaction, leading to a long-term structural stability and improved cyclability, while their end-members transform through a two-phase reaction [24,25,26]. LiFePO_4_F, LiVPO_4_F and LiVPO_4_O have homotypic structures. We have reported recently the LiFePO_4_F–LiVPO_4_O solid solutions (i.e., LiFe_1−*x*_V*_x_*PO_4_F_1−_*_δ_*O*_δ_* (0 ≤ *x* ≤ 1; 0 ≤ *δ* ≤ 0.36)) [26]. Otherwise, there are some publications related to LiVPO_4_F–LiVPO_4_O [27,28,29,30,31] and a few to LiFePO_4_F–LiVPO_4_F [25,32] solid solutions. Not much information can be collected from the meeting abstract [32]. Huang et al. [25] synthesized LiFe_0.5_V_0.5_PO_4_F solid-solution which showed a single-phase behavior over the lithium composition range of Li_1−_*_y_*Fe_0.5_V_0.5_PO_4_F (0 < *y* < 0.5) with two alternating electrochemical active regions centered at ~2.76 and ~4.3 V.

In this work, LiFe_1−*x*_V*_x_*PO_4_F (*x* = 0, 0.1, 0.3, 0.5, 0.7, 0.9, 1) powders, cathodes and the corresponding Li-ion batteries were prepared and characterized. The object is to study the two- to single-phase Li^+^-extraction/insertion mechanism through tuning the transition-metal stoichiometry of cations to trigger a transition in the chemical reactivity path.

## 2. Results

### 2.1. Phase Structure

**Figure 1:**
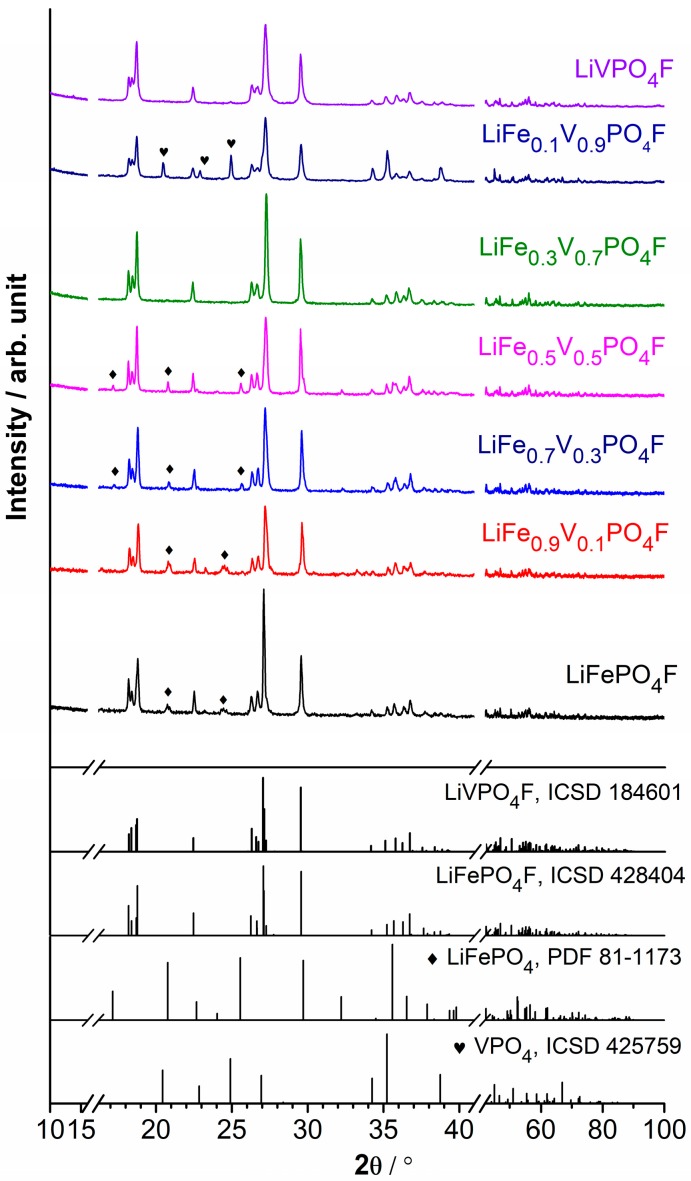
XRD full patterns of LiFe_1−*x*_V*_x_*PO_4_F (0 ≤ *x* ≤ 1) powders.

Figure 1 shows XRD full patterns of LiFe_1−*x*_V*_x_*PO_4_F (0 ≤ *x* ≤ 1) powders. Tables S1–S7 list Rietveld refined parameters of the corresponding tavorite structures. Table S8 shows comparison of lattice parameters for LiFe_1−*x*_V*_x_*PO_4_F (0 ≤ *x* ≤ 1) samples and the related publications. Figure S1 shows the final observed, calculated and difference profiles of the tavorite-structured LiFePO_4_F, LiFe_0.9_V_0.1_PO_4_F, LiFe_0.7_V_0.3_PO_4_F, LiFe_0.5_V_0.5_PO_4_F, LiFe_0.3_V_0.7_PO_4_F, LiFe_0.1_V_0.9_PO_4_F and LiVPO_4_F via Rietveld refinements. Figure S2 shows variations of lattice parameters and unit cell volumes of LiFe_1−*x*_V*_x_*PO_4_F (0 ≤ *x* ≤ 1) solid solutions. Further details of crystal structures may be obtained from the website listed in Appendix A. Under harsh testing conditions, pure LiVPO_4_F and LiFe_0.3_V_0.7_PO_4_F phases have been attained. There are a few LiFePO_4_ impurities (~1.7, ~2.4, ~4.1 and 6.3% wt, respectively) when *x* = 0, 0.1, 0.3 and 0.5, and VPO_4_ impurity (~7.5% wt) when *x* = 0.9 in LiFe_1−*x*_V*_x_*PO_4_F (0 ≤ *x* ≤ 1) samples, which will be further discussed in electrochemical measurements (Section 2.4).

Considering the crystal structures are triclinic, the continuous substitutions lead to a versatile change of interplanar crystal spacing. Refinements reveal that solid-solution domains exist without phase separation [25,32]. Lattice parameters (***a***, ***b***, ***c***, α, β, γ) and unit cell volumes (*V*) of end-members (*x* = 0 and 1) agree well with our previous results [4,26,33]. There is only one crystallographic Li site (2*i*) [7,11] and two independent Fe/V sites (1*a* and 1*c*) in the unit cell. The *M*–O_4_F_2_ chains (*M* = Fe_1−*x*_V*_x_*) along the *b* axis constitute an alternation of *M*1 and *M*2 centered octahedra which are slightly distorted. The F ligands (2*i*) act as the bridging ligands. Each oxygen (2*i*) from the equatorial plane of the octahedron is common to a PO_4_ tetrahedron bridging the *M*–O_4_F_2_ chains, leading to the formation of a 3D framework. The systematic variations in lattice parameters and unit cell volumes of LiFe_1−*x*_V*_x_*PO_4_F (0 < *x* < 1) samples confirm the formation of homogeneous solid solutions, which originate from substitutions by the V^3+^ ($r_{V^{3+}}$ = 0.640 Å) for Fe^3+^ ($r_{{\mathrm{Fe}}^{3+},\mathrm{HS}}$ = 0.645 Å in a high spin (HS) state) with close effective ionic radii while the coordination number (CN) is 6 [3,34,35]. The published unit cell volumes *V* (Å^3^) of the related phases are collected as follows: LiFePO_4_F (173.91(2) [22], 173.67(6) [23], 173.558(6) [4]), LiVPO_4_F (174.36(2) [11], 174.31 [36], 174.25(1) [30], 174.167(16) [33]) and LiVPO_4_O (171.018(1) [11], 171.227(2) [37], 171.578(3) [38]). Therefore, the volume deviation (Δ*V*) between LiFePO_4_F and LiVPO_4_F is under 0.47%, much less than that between LiFePO_4_F and LiVPO_4_O (under 1.7%). For example, the *V* value (173.25(1) Å^3^) of LiFe_0.5_V_0.5_PO_4_F (the only LiFePO_4_F–LiVPO_4_F solid-solution reported) is not located between end-members thus against the Vegard’s law [25]. It may be caused by experiment errors because of the small volume deviation. However, the *V* values of LiFePO_4_F–LiVPO_4_O solid solutions are located between end-members [26]. In this work, the *V* values of the prepared LiFePO_4_F–LiVPO_4_F samples are located in a narrow region (0.33–0.85%; Figure S2) due to the close effective ionic radii of Fe^3+^ (0.645 Å) and V^3+^ (0.640 Å), indicating the formation of solid solutions.

### 2.2. Powder Microstructure

**Figure 2:**
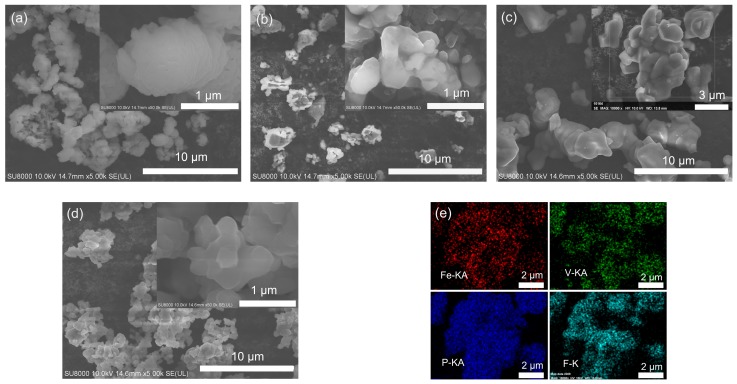
SEM images of FePO_4_ (**a**), VPO_4_ (**b**), LiFe_0.5_V_0.5_PO_4_F (**c**) and LiVPO_4_F (**d**), and the EDS mapping of LiFe_0.5_V_0.5_PO_4_F (**e**) from the inset in Figure 2c.

Figure 2 shows SEM images of FePO_4_, VPO_4_, LiFe_0.5_V_0.5_PO_4_F and LiVPO_4_F, and the energy dispersive spectra (EDS) mapping of LiFe_0.5_V_0.5_PO_4_F.

The particle size is 1–2 μm for FePO_4_ (Figure 2a), 0.5–1 μm for VPO_4_ (Figure 2b), 1–2 μm for LiFe_0.5_V_0.5_PO_4_F (Figure 2c) and 0.5–1.5 μm for LiVPO_4_F (Figure 2d). The EDS test confirms a nearly-nominal proportion (Fe:V:P = 0.46:0.57:1, %mol) and a homogeneous distribution of Fe, V, P and F components in the LiFe_0.5_V_0.5_PO_4_F solid solutions (Figure 2e), indicating that substitutions are successful.

### 2.3. Valence States of Fe/V Components

**Figure 3:**
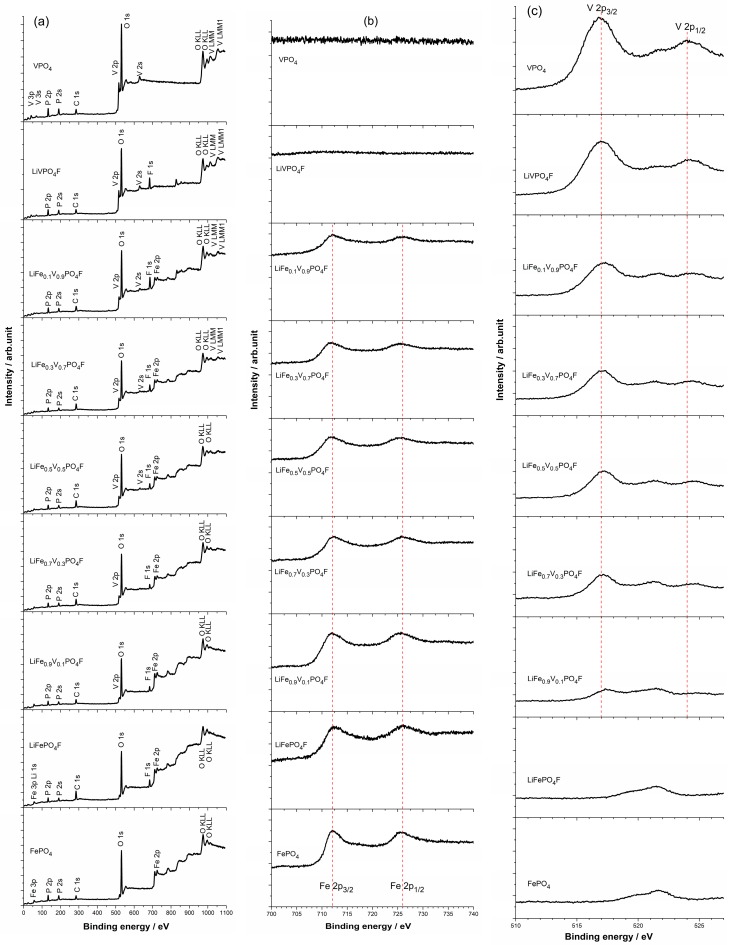
X-ray photoelectron spectra (XPS) of FePO_4_, VPO_4_ and LiFe_1−*x*_V*_x_*PO_4_F (0 ≤ *x* ≤ 1) powders (**a**). Binding energy regions of the Fe 2p (**b**) and V 2p (**c**) show spin-orbit splitting of 2p_3/2_ and 2p_1/2_, respectively.

The core level X-ray photoelectron spectra (XPS) of FePO_4_, VPO_4_ and LiFe_1−*x*_V*_x_*PO_4_F (0 ≤ *x* ≤ 1) powders are shown in Figure 3a. The Fe 2p (or V 2p) spectrum consists of two components (Fe 2p_3/2_/Fe 2p_1/2_ or V 2p_3/2_/V 2p_1/2_) due to spin-orbit (*j*–*j*) coupling/splitting (Figure 3b,c) [39,40].

In Figure 3b, main peaks of Fe 2p_3/2_ and Fe 2p_1/2_ centered respectively at ~712/726 eV are assigned to the high-spin Fe^3+^ species with the (3d↑)^5^(3d↓)^0^ electronic configuration [41], similar to the reported FePO_4_ (712.5/726 eV [39,42]). The Fe 2p_3/2_ peak is narrower and stronger than Fe 2p_1/2_, and the area of Fe 2p_3/2_ is greater than that of Fe 2p_1/2_ because 2p_3/2_ has the degeneracy of four multiplets while 2p_1/2_ has only two in *j*–*j* coupling [40,41]. By contrast, for a high-spin Fe^2+^ species like LiFePO_4_ with (3d↑)^5^(3d↓)^1^ configuration, main peaks of Fe 2p_3/2_ and Fe 2p_1/2_ are centered at 710.5/724 eV, respectively [39,42].

In Figure 3c, main peaks of V 2p_3/2_ and V 2p_1/2_ centered respectively at ~517/524 eV are assigned to the V^3+^ species with the (3d↑)^2^(3d↓)^0^ electronic configuration [43], similar to the reported VPO_4_ (517.3/524.8 eV [44]) and LiVPO_4_F (517.3/524.7 eV [44]; 517.2/523.0 eV [45]; 517.1/523.4 eV [46]; 517.38/524.81 eV [31]). By contrast, for a V^4+^ species like LiVPO_4_O with (3d↑)^1^(3d↓)^0^ configuration, main peaks of V 2p_3/2_ and V 2p_1/2_ are centered at 518.09/525.38 eV, respectively [31]. It can be concluded then that valence states of Fe and V are +3 in FePO_4_, VPO_4_ and LiFe_1−*x*_V*_x_*PO_4_F (0 ≤ *x* ≤ 1) samples.

### 2.4. Shift in Redox Potential

**Figure 4:**
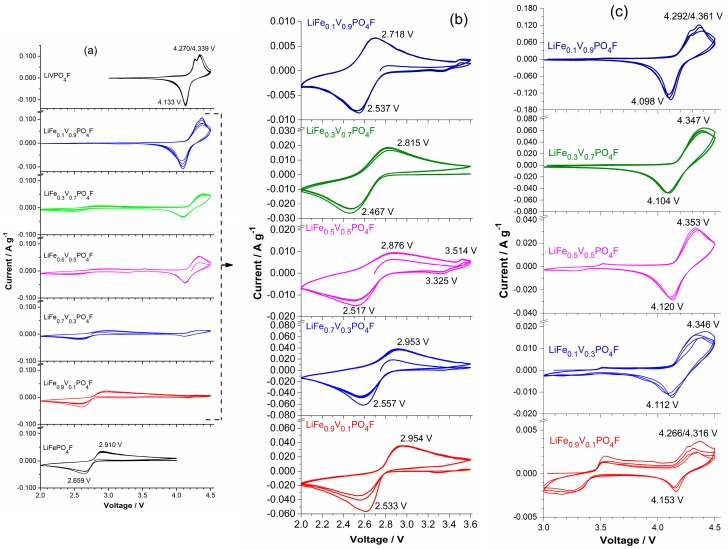
Cyclic voltammetry (CV) curves of LiFe_1−*x*_V*_x_*PO_4_F (*x* = 0, 0.1, 0.3, 0.5, 0.7, 0.9, 1) cells with the same sweep rate of 0.1 mV s^−1^ for five cycles in the range of 2.0–4.5 V (**a**), 2.0–3.6 V (**b**) and 3.0–4.5 V (**c**). With the exception of the starting half cycle, all of peak positions are from the second cycle.

**Figure 5:**
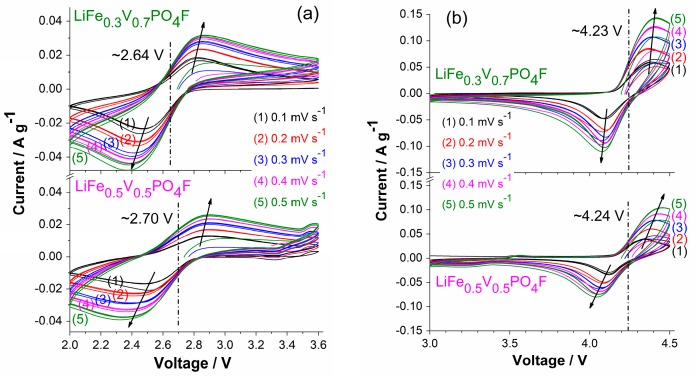
CV curves of LiFe_0.5_V_0.5_PO_4_F and LiFe_0.3_V_0.7_PO_4_F cells with different sweep rates of 0.1/0.2/0.3/0.4/0.5 mV s^−1^ for 15 cycles in the range of 2.0–3.6 V (**a**) and 3.0–4.5 V (**b**).

**Figure 6:**
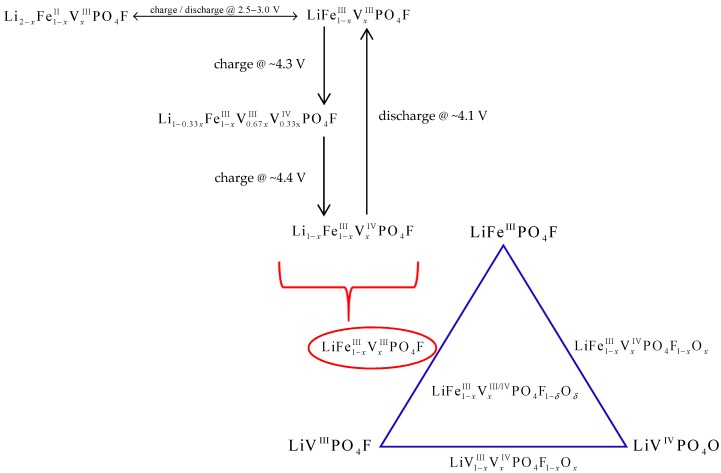
Total reactions in LiFe_1−*x*_V*_x_*PO_4_F (0 ≤ *x* ≤ 1) cells.

Figure 4 shows cyclic voltammetry (CV) curves of LiFe_1−*x*_V*_x_*PO_4_F (*x* = 0, 0.1, 0.3, 0.5, 0.7, 0.9, 1) cells with the same sweep rate of 0.1 mV s^−1^ for five cycles. Figure 5 shows CV curves of LiFe_0.5_V_0.5_PO_4_F and LiFe_0.3_V_0.7_PO_4_F cells with different sweep rates of 0.1/0.2/0.3/0.4/0.5 mV s^−1^ for 15 cycles. Figure 6 shows total reactions in LiFe_1−*x*_V*_x_*PO_4_F (0 ≤ *x* ≤ 1) cells. The CV data of LiFePO_4_F are consistent with our previous results [4,26] and that of others [1], in which a pair (cathodic/anodic) of redox peaks exist at 2.659/2.910 V assigned to the Fe^2+/3+^ couple for ${\mathrm{Li}}_{2}{\mathrm{Fe}}^{\mathrm{II}}{\mathrm{PO}}_{4}F$/${\mathrm{LiFe}}^{\mathrm{III}}{\mathrm{PO}}_{4}F$. The CV data of LiVPO_4_F are in good agreement with results of the reported differential capacity vs. voltage curves [6,47,48] and CV tests [44,49]. Split anodic peaks at 4.270/4.339 V ascribes to the occurrence of an intermediate phase (${\mathrm{Li}}_{0.67}{\mathrm{VPO}}_{4}F$, i.e., ${\mathrm{Li}}_{0.67}V_{0.67}^{\mathrm{III}}V_{0.33}^{\mathrm{IV}}{\mathrm{PO}}_{4}F$) during oxidation (Li^+^-extraction), reflecting two energetically inequivalent reactions (${\mathrm{LiV}}^{\mathrm{III}}{\mathrm{PO}}_{4}F$ $\overset{\;\mathrm{charge}\;@\;4.270\;V\;}{\to }$ ${\mathrm{Li}}_{0.67}V_{0.67}^{\mathrm{III}}V_{0.33}^{\mathrm{IV}}{\mathrm{PO}}_{4}F$ $\overset{\;\mathrm{charge}\;@\;4.339\;V\;}{\to }V^{\mathrm{IV}}{\mathrm{PO}}_{4}F$). The corresponding structure evolution is: triclinic *P*$\bar{1}$ → triclinic *P*$\bar{1}$ → monoclinic *C*2/*c* [7,11]. A single cathodic peak at 4.133 V characterizes a two-phase Li^+^-insertion process ($V^{\mathrm{IV}}{\mathrm{PO}}_{4}F\;\overset{\;\mathrm{discharge}\;@\;4.133\;V\;}{\to }{\mathrm{LiV}}^{\mathrm{III}}{\mathrm{PO}}_{4}F$). The corresponding structure evolution is: monoclinic *C*2/*c* → triclinic *P*$\bar{1}$ [6,44,47,48,49].

For LiFe_1−*x*_V*_x_*PO_4_F samples (0 < *x* < 1), there still exists a pair of redox peaks assigned to the Fe^2+/3+^ couple in which systematic shifts of cathodic/anodic peaks were observed. This will be discussed in detail later. There also exists two anodic peaks (or overlapping peaks while *x* = 0.3, 0.5 and 0.7) at 4.266–4.361 V similar to LiVPO_4_F, indicating the occurrence of intermediate phases (${\mathrm{Li}}_{1-0.33x}{\mathrm{Fe}}_{1-x}^{\mathrm{III}}V_{0.67x}^{\mathrm{III}}V_{0.33x}^{\mathrm{IV}}{\mathrm{PO}}_{4}F$, triclinic, *P*$\bar{1}$) [7,11]. There is a single cathodic peak at 4.126 ± 0.027 V which characterizes a two-phase Li^+^-insertion process (${\mathrm{Li}}_{1-x}{\mathrm{Fe}}_{1-x}^{\mathrm{III}}V_{x}^{\mathrm{IV}}{\mathrm{PO}}_{4}F\overset{\;\mathrm{discharge}\;@\;4.126\;\pm \;0.027\;V\;}{\to }{\mathrm{LiFe}}_{1-x}^{\mathrm{III}}V_{x}^{\mathrm{III}}{\mathrm{PO}}_{4}F$). The corresponding structure evolution is: monoclinic *C*2/*c* → triclinic *P*$\bar{1}$. Important to note is the shape of CV peaks. The broad shape of CV peaks for LiFe_1−*x*_V*_x_*PO_4_F solid-solution samples while 0 < *x* < 1, instead of the sharp and narrow peaks as observed in end-members (*x* = 0 or 1), is indicative of a single-phase solid-solution behavior [7,25]. Otherwise, the pair of redox peaks at ~3.35/3.55 V when *x* = 0.1, 0.3 and 0.5 are assigned to the Fe^2+/3+^ couple for LiFePO_4_ impurity (Figure 4b,c), consistent with the XRD results (Figure 1). Note that the current (A g^−1^) for Fe^2+/3+^ couple in LiFePO_4_ impurity is one order of magnitude smaller than that for Fe^2+/3+^ couple in LiFe_0.9_V_0.1_PO_4_F sample, indicating that the impurity content is very small while the V-doping amount is low (Figure 4c). The above electrochemical reactions can be summarized as the following:

(1) $${\mathrm{LiFeV}}_{1-x}^{\mathrm{III}}V_{x}^{\mathrm{III}}{\mathrm{PO}}_{4}F\;\overset{\;\mathrm{charge}\;@\;\sim 4.3\;V\;}{\to }{\mathrm{Li}}_{1-0.33x}{\mathrm{Fe}}_{1-x}^{\mathrm{III}}V_{0.67x}^{\mathrm{III}}V_{0.33x}^{\mathrm{IV}}{\mathrm{PO}}_{4}F+0.33x{\mathrm{Li}}^{+}+0.33xe^{-},$$

(2) $${\mathrm{Li}}_{1-0.33x}{\mathrm{Fe}}_{1-x}^{\mathrm{III}}V_{0.67x}^{\mathrm{III}}V_{0.33x}^{\mathrm{IV}}{\mathrm{PO}}_{4}F\;\overset{\;\mathrm{charge}\;@\;\sim 4.4\;V\;}{\to }{\mathrm{Li}}_{1-x}{\mathrm{Fe}}_{1-x}^{\mathrm{III}}V_{x}^{\mathrm{IV}}{\mathrm{PO}}_{4}F+0.67x{\mathrm{Li}}^{+}+0.67xe^{-},$$

(3) $${\mathrm{Li}}_{1-x}{\mathrm{Fe}}_{1-x}^{\mathrm{III}}V_{x}^{\mathrm{IV}}{\mathrm{PO}}_{4}F+x{\mathrm{Li}}^{+}+xe^{-}\;\overset{\;\mathrm{discharge}\;@\;\sim 4.1\;V\;}{\to }{\mathrm{LiFe}}_{1-x}^{\mathrm{III}}V_{x}^{\mathrm{III}}{\mathrm{PO}}_{4}F,$$

(4) $${\mathrm{LiFe}}_{1-x}^{\mathrm{III}}V_{x}^{\mathrm{III}}{\mathrm{PO}}_{4}F+\left(1-x\right){\mathrm{Li}}^{+}+\left(1-x\right)e^{-}\overset{\;\mathrm{discharge}\;@\;2.5–3.0\;V\;}{↔}{\mathrm{Li}}_{2-x}{\mathrm{Fe}}_{1-x}^{\mathrm{II}}V_{x}^{\mathrm{III}}{\mathrm{PO}}_{4}F,$$

Therefore, whether LiFe_1−_*_x_*V*_x_*PO_4_F cells (0 ≤ *x* ≤ 1) cycled in the range of 2.0–4.5 V charge firstly and discharge subsequently, or vice versa, total reactions can be concluded shown in Figure 6. Now the end-member phases change from LiFePO_4_F/LiVPO_4_F to ${\mathrm{Li}}_{2-x}{\mathrm{Fe}}_{1-x}^{\mathrm{II}}V_{x}^{\mathrm{III}}{\mathrm{PO}}_{4}F/{\mathrm{Li}}_{1-x}{\mathrm{Fe}}_{1-x}^{\mathrm{III}}V_{x}^{\mathrm{IV}}{\mathrm{PO}}_{4}F$.

When LiFe_1−_*_x_*V*_x_*PO_4_F (*x* = 0, 0.1, 0.3, 0.5, 0.7, 0.9, 1) cells cycle at 0.1 mV s^−1^ (Figure 4), or cycle respectively at 0.2, 0.3, 0.4 and 0.5 mV s^−1^, the cathodic/anodic peaks remain unmoved and peak areas increase, indicating that they have good structural stability and good cyclability (Figure 5). But cathodic/anodic peaks shift during sweep rates changing. When cells cycle from a lower rate to a higher one (0.1 → 0.2 → 0.3 → 0.4 → 0.5 mV s^−1^), cathodic peaks shift to lower potentials and the corresponding anodic peaks shift to higher potentials. Simultaneously, the potential differences (Δ*E_p_*) increase from 0.36 V to 0.47 V for Fe^2+/3+^ couple and from 0.23 V to 0.44 V for V^3+/4+^ couple in the LiFe_0.5_V_0.5_PO_4_F cell. Additionally, Δ*E_p_* increases from 0.35 V to 0.53 V for Fe^2+/3+^ couple and from 0.25 V to 0.32 V for V^3+/4+^ couple in the LiFe_0.3_V_0.7_PO_4_F cell. This is due to electrode kinetics or electrode polarization related to the formation of SEI (solid electrolyte interface) film, side reactions, capacity-fading, etc. Slight differences in shapes of anodic/cathodic peaks are due to cycle reforming [1,4,26,50].

**Figure 7:**
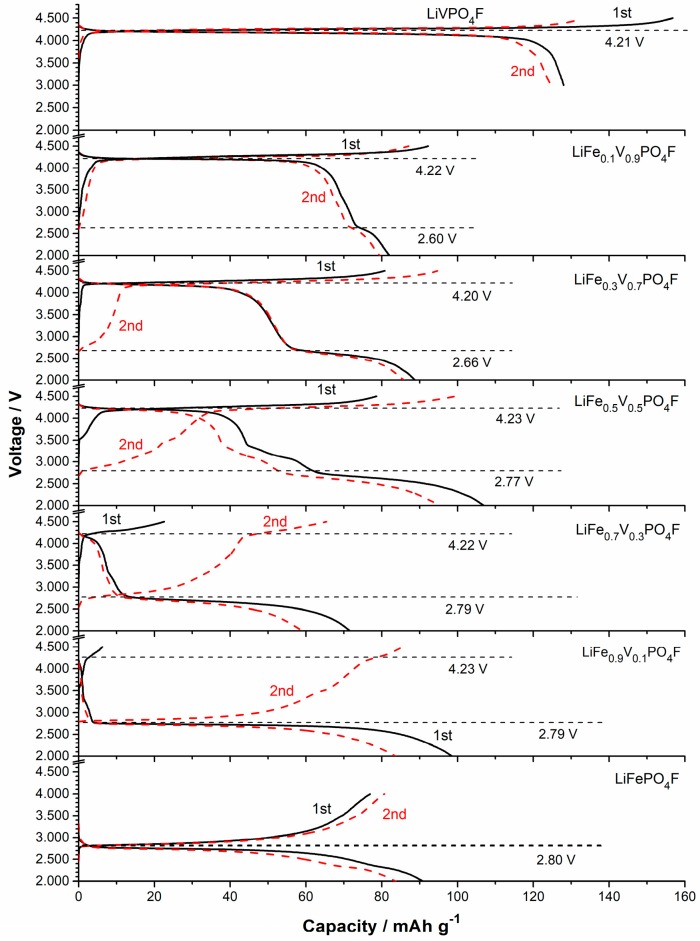
The initial and second charge/discharge profiles of LiFe_1−*x*_V*_x_*PO_4_F (*x* = 0, 0.1, 0.3, 0.5, 0.7, 0.9, 1) cells at 0.1 C.

Galvanostatic charge/discharge tests are to understand redox couples and examine the presence of multiple phases. Figure 7 shows the initial and second charge/discharge profiles of LiFe_1−*x*_V*_x_*PO_4_F (0 ≤ *x* ≤ 1) cells at 0.1 C. The galvanostatic tests do not show two separate voltage plateaus on charges around 2.0–4.5 V because of their higher scan rate (0.1 C) than the reported (0.02 C [7,11]), and the lower resolution than CV tests (Figure 4 and Figure 5). A flat plateau at ~2.7 V is assigned to Fe^2+/3+^ couple and the plateau at ~4.2 V to V^3+/4+^ couple. The additional redox plateau at ~3.4 V in LiFe_0.5_V_0.5_PO_4_F sample is assigned to Fe^2+/3+^ couple for LiFePO_4_ impurity, consistent with the XRD and CV results (Figure 1 and Figure 4). Noteworthy is the Li^+^-extraction/insertion behavior. In regions of 2.0–3.0 V and 3.0–4.5 V, sloping charge/discharge profiles were observed for all of the LiFe_1−*x*_V*_x_*PO_4_F solid-solution samples while 0 < *x* < 1. This indicates a single-phase behavior [24,25,26,51] which is different from the two-phase behavior for the end-members (*x* = 0 or 1).

### 2.5. Li-Ion Diffusion Behavior

**Figure 8:**
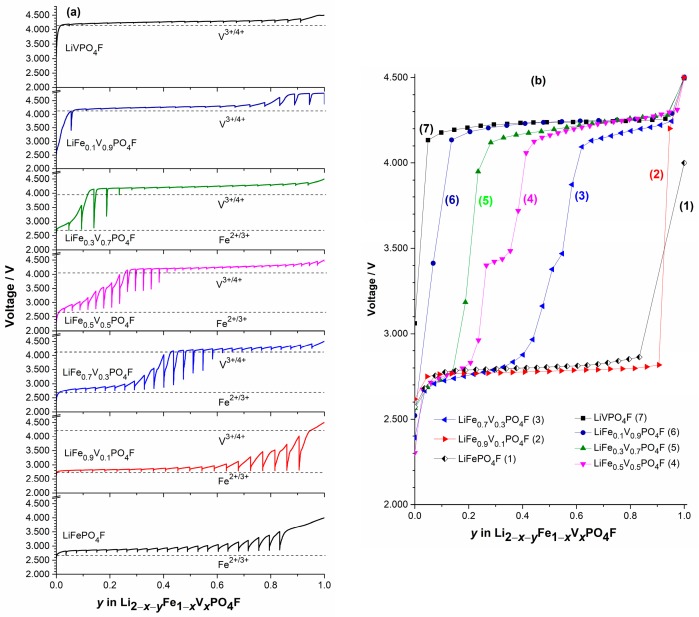
The independent (**a**) and overlaid (**b**) curves of voltage as a function of Li^+^-extraction content *y* under load and rest by galvanostatic intermittent titration technique (GITT) measurements in ${\mathrm{Li}}_{2-x-y}{\mathrm{Fe}}_{1-x}^{\mathrm{II}}V_{x}^{\mathrm{III}}{\mathrm{PO}}_{4}F$ (0 ≤ *x* ≤ 1; 0 ≤ *y* ≤ 1).

**Figure 9:**
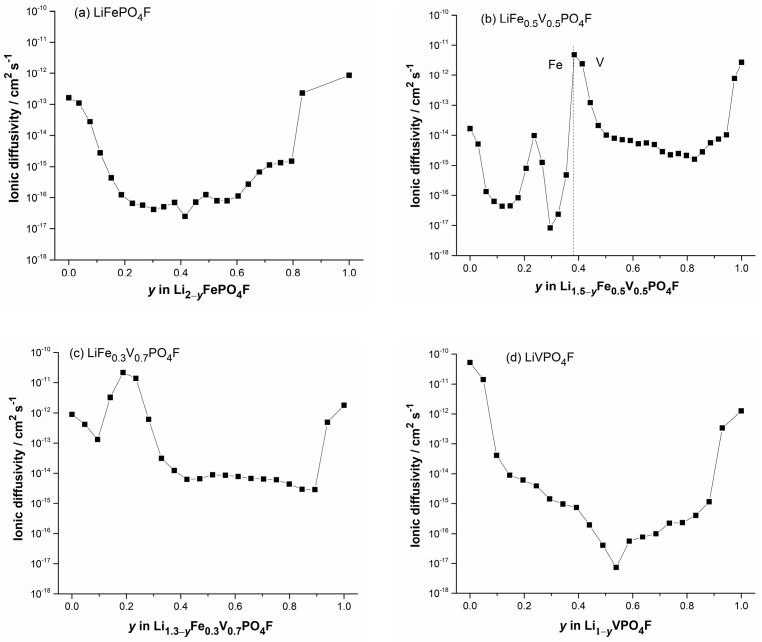
Plots of diffusion coefficients obtained by GITT as a function of Li^+^-extraction content *y* in ${\mathrm{Li}}_{2-x-y}{\mathrm{Fe}}_{1-x}^{\mathrm{II}}V_{x}^{\mathrm{III}}{\mathrm{PO}}_{4}F$ (0 ≤ *y* ≤ 1) with *x* = 0 (**a**), *x* = 0.5 (**b**), *x* = 0.7 (**c**) and *x* = 1 (**d**).

To fully understand the electrochemical reactions occurring during Li^+^-extraction/insertion process in LiFe_1−*x*_V*_x_*PO_4_F (0 ≤ *x* ≤ 1) cathodes, galvanostatic intermittent titration technique (GITT) measurements were carried out to evaluate the Li-ion diffusion behavior (Figure 8 and Figure 9). Figure S3 shows a scheme for a GITT measurement.

Diffusion coefficient of lithium ions ($D_{{\mathrm{Li}}^{+}}$, in cm^2^ s^−1^) is calculated based on Equation (5) derived by Weppner et al. [52]:

(5) $$D_{{\mathrm{Li}}^{+}}=4/\pi {\left(V_{M}/\left(SF\right)\right)}^{2}{\left(I_{0}\left(\delta E_{s}/y\right)/\left(\delta E/\delta t^{1/2}\right)\right)}^{2}\;at\;t<<\tau ,$$

where *S* is the contact area between the sample and electrolyte (cm^2^ g^−1^). In this work, it is calculated from the mean diameter of approximately-spherical grains determined by SEM (Figure 2). The corresponding *S* values of samples are 8.87 × 10^4^ (LiFePO_4_F), 1.80 × 10^4^ (LiFe_0.5_V_0.5_PO_4_F), 1.81 × 10^4^ (LiFe_0.3_V_0.7_PO_4_F) and 3.66 × 10^4^ cm^2^ g^−1^ (LiVPO_4_F), respectively. This is a suitable choice since *c.f.* errors would be introduced from the residual carbon when using the Brunauer–Emmett–Teller (BET) specific surface area, or from the cathode-in-electrolyte system when using the electrode geometric area. Thus, calculation results may cause a big difference on about several orders of magnitude, but the general trend of $D_{{\mathrm{Li}}^{+}}$ will be the same [53,54,55]. *V_M_* is the molar volume of sample (cm^3^ mol^−1^), *F* is the Faraday constant (9.64853 × 10^4^ C mol^−1^), and *I*_0_ is the pulse current (A g^−1^). $\delta E_{s}/\delta y$ is the slope of quasi-equilibrium open-circuit voltage (OCV) (V) as a function of Li^+^-extraction content *y*. $\delta E/\delta t^{1/2}$ is the slope of the initial transient voltage change as a function of the square root of time (V s^−1/2^). The equation is valid for times shorter than the diffusion time $\tau ={\left(\pi d/2\right)}^{2}/D_{{\mathrm{Li}}^{+}}$, where *d* is the average diameter of grains [54]. If the arithmetical units of $D_{{\mathrm{Li}}^{+}}$ were in m^2^ s^−1^, those of *S* should be in m^2^ g^−1^ and *V_M_* in m^3^ mol^−1^ simultaneously.

Figure 8 shows the independent (Figure 8a) and overlaid (Figure 8b) curves of voltage as a function of Li^+^-extraction content *y* under load and rest by GITT measurements in ${\mathrm{Li}}_{2-x-y}{\mathrm{Fe}}_{1-x}^{\mathrm{II}}V_{x}^{\mathrm{III}}{\mathrm{PO}}_{4}F$ (0 ≤ *x* ≤ 1; 0 ≤ *y* ≤ 1). Here, the nearly flat region indicates the voltage measured during charging (load), while relaxation spikes at a given state of charge (SOC) (i.e., Li^+^-extraction content *y*) indicate the change in voltage during relaxation or equilibration. The equilibrium OCVs for Fe^2+/3+^ couples in LiFePO_4_F and V^3+/4+^ couples in LiVPO_4_F reveal nearly flat potentials on 2.78 V and 4.24 V, respectively. This means a two-phase reaction mechanism. The LiFePO_4_F sample exhibits higher over-voltage (longer spikes) than the LiVPO_4_F, indicating its larger polarization and slower equilibration [56]. The OCV profiles of LiFe_1−*x*_V*_x_*PO_4_F solid-solution samples (0 < *x* < 1), especially for those with *x* = 0.3, 0.5 and 0.7, show a sloping region on going from Fe^2+/3+^ to V^3+/4+^ redox couples. This indicates a single-phase reaction mechanism [25,32,56].

Figures S4–S7 show GITT curves of the quasi-equilibrium OCVs as a function of time, or as a function of Li^+^-extraction content *y*, plots of the slope of quasi-equilibrium OCVs as a function of Li^+^-extraction content *y* ($\delta E_{s}/\delta y$), and plots of the slope of initial transient voltage change as a function of the square root of time ($\delta E/\delta t^{1/2}$), in ${\mathrm{Li}}_{1-y}{\mathrm{Fe}}_{1-x}^{\mathrm{III}}V_{x}^{\mathrm{III}}{\mathrm{PO}}_{4}F$ (i.e., ${\mathrm{Li}}_{2-x-y}{\mathrm{Fe}}_{1-x}^{\mathrm{II}}V_{x}^{\mathrm{III}}{\mathrm{PO}}_{4}F$ with *x* = 0, 0.5, 0.7, 1). Figure 9 shows plots of diffusion coefficients $D_{{\mathrm{Li}}^{+}}$ obtained by GITT as a function of Li^+^-extraction content *y*. They present disordered “W” or “U” shapes for extraction/insertion, similar to those reported [53].

The obtained $D_{{\mathrm{Li}}^{+}}$ values vary from ~10^−17^ to ~10^−12^ cm^2^ s^−1^ (LiFePO_4_F), ~10^−17^ to 10^−11^ cm^2^ s^−1^ (LiFe_0.5_V_0.5_PO_4_F), ~10^−15^ to ~5 × 10^−11^ cm^2^ s^−1^ (LiFe_0.3_V_0.7_PO_4_F) and ~10^−^^17^ to ~10^–10^ cm^2^ s^−1^ (LiVPO_4_F), respectively. Each of them has a minimum which was caused by strong attractive interactions between the Li^+^-extraction/insertion species and the host matrix [54]. As the doped V-content *x* increases from 0 to 1, the upper-limit value of $D_{{\mathrm{Li}}^{+}}$ increases 1–2 orders of magnitude. If the geometric area of the electrode was used as the contact area (*S*) [25,55], diffusion coefficients would increase 2–3 orders of magnitude for samples in this work. It indicates that LiFe_1−*x*_V*_x_*PO_4_F (0 < *x* < 1) solid solutions have comparable electrochemical activities with their end-members (*x* = 0 or 1), while redox potentials can be tuned within a wide range, 2.0–4.5 V, by cation (V for Fe or Fe for V) substitutions. This makes them attractive cathode candidates of high specific-energy Li-ion batteries.

## 3. Discussion

**Figure 10:**
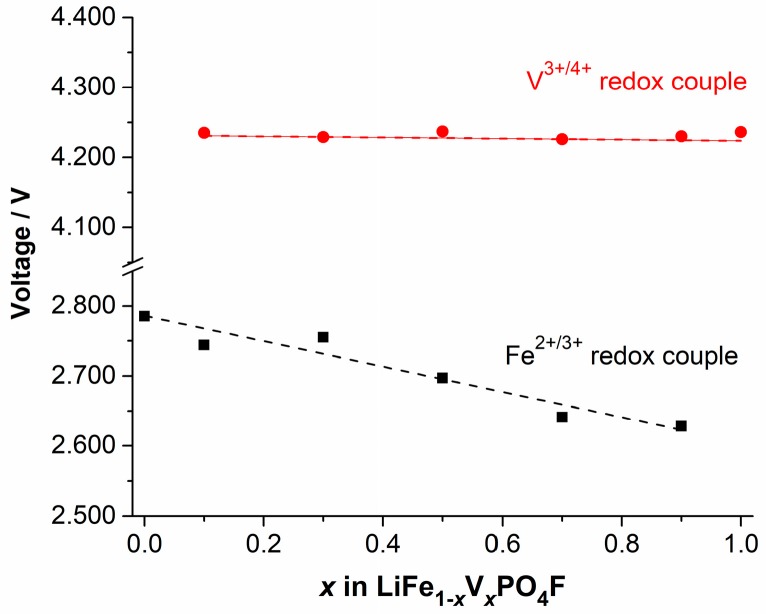
Shifts in midpoints of anodic (Li^+^-extraction) and cathodic (Li^+^-insertion) peaks for Fe^2+/3+^ and V^3+/4+^ couples, exported from Figure 4, as a function of V-content *x* in LiFe_1−*x*_V*_x_*PO_4_F (0 ≤ *x* ≤ 1).

Figure 10 shows shifts in midpoints of anodic (Li^+^-extraction) and cathodic (Li^+^-insertion) peaks for Fe^2+/3+^ and V^3+/4+^ couples, exported from Figure 4, as a function of V-content *x* in LiFe_1−*x*_V*_x_*PO_4_F (0 ≤ *x* ≤ 1). As mentioned in Section 2.4, there exists two anodic peaks assigned to V^3+/4+^ redox couple when 0 < *x* ≤ 1, and the separation of anodic peaks is ~0.07 V. For simplicity, one midpoint was calculated from a cathodic peak and its corresponding higher-potential anodic peak. As the V-content *x* increases, a downward shift in the redox potential of Fe^2+/3+^ couple was observed. However, there was hardly any shift for V^3+/4+^ couple. These are different significantly from those in the reported $\mathrm{Li}M_{1-y}^{\prime }M_{y}^{\prime }{\mathrm{PO}}_{4}$ (*M′*, *M″* = Mn, Fe, Co), in which potentials increasing of lower potential (LP)-couples is always associated with potentials decreasing of high potential (HP)-couples [56,57,58], compared to potentials of the pristine end-members.

Redox energies of cations can be tuned through the inductive effect introduced by a counter cation substitution [24,25,56,59] in ${\mathrm{LiFe}}_{1-x}^{\mathrm{III}}V_{x}^{\mathrm{III}}{\mathrm{PO}}_{4}F$ (0 < *x* < 1) solid-solution cathodes. If the polyanion (PO_4_F) was fixed, the change in the covalency of *M*–O_4_F_2_ bonds (*M* = Fe_1−*x*_V*_x_*) could be caused by the following:

(i) Change in the electronegativity of *M*: The substitution of a less electronegative (more electropositive) V^3+^ for Fe^3+^ is expected to increase the Fe–O_4_F_2_ covalency due to the inductive effect (weaker V–O_4_F_2_ covalency strengthens the Fe–O_4_F_2_ covalency), and raise the Fe^2+/3+^ redox energy, thereby decreasing the redox potential of Fe^2+/3+^ couple, in accord with what we observe in Figure 10. Similarly, the substitution of a more electronegative Fe^3+^ for V^3+^ would be expected to decrease the V–O_4_F_2_ covalency, lower the V^3^^+/4+^ redox energy, and increase the redox potential of the V^3^^+/4+^ couple [56,57,58]. This is not in accord with what we observe in Figure 10.

(ii) Change in the *M*–O_4_F_2_ bond length: The covalency contraction effect originates from the relative contraction of cation-anion distances in two different isotypic compounds with different electronegativity [35]. As stated before, the V^3+^ ($r_{V^{3+}}$ = 0.640 Å) and Fe^3+^ ($r_{{\mathrm{Fe}}^{3+},\mathrm{HS}}$ = 0.645 Å) have close effective ionic radii (CN = 6) [3,34,35]. In a high spin (HS) state, the covalency contraction effect and crystal field effect play collectively a dominant role on the Fe^2+^ ($r_{{\mathrm{Fe}}^{2+},\mathrm{HS}}$ = 0.780 Å) and Fe^3+^ ($r_{{\mathrm{Fe}}^{3+},\mathrm{HS}}$ = 0.645 Å), and as a result their radii are close to the V^2+^ ($r_{V^{2+}}$ = 0.79 Å) and V^3+^ ($r_{V^{3+}}$ = 0.640 Å), respectively. But in a low spin (LS) state, the covalency contraction effect plays a dominant role on the Fe^2+^ ($r_{{\mathrm{Fe}}^{2+},\mathrm{LS}}$ = 0.61 Å) and Fe^3+^ ($r_{{\mathrm{Fe}}^{3+},\mathrm{LS}}$ = 0.55 Å), and as result their radii are smaller than the V^2+^ ($r_{V^{2+}}$ = 0.79 Å) and V^3+^ ($r_{V^{3+}}$ = 0.640 Å), respectively [3,34,35]. The substitution of V^3+^ ($r_{V^{3+}}$ = 0.640 Å) for Fe^3+^ ($r_{{\mathrm{Fe}}^{3+},\mathrm{HS}}$ = 0.645 Å) with close effective ionic radii does not change the *M*–O_4_F_2_ bond length (therefore it does not change the Fe–O_4_F_2_ or V–O_4_F_2_ covalency), indicating that redox energies/potentials of Fe^2+/3+^ and V^3^^+/4+^ couples would not change which correlates to the inductive effect [56,57,58]. This identifies with what we observe in Figure 10. We do not support that the Fe^3+/4+^ couple exists stably in range of 2.0–4.5 V [4,26,60].

Therefore, we can conclude that the electronegativity of *M* plays a dominant role compared to the *M*–O_4_F_2_ bond length for the redox potential of Fe^2+/3+^ couple in ${\mathrm{LiFe}}_{1-x}^{\mathrm{III}}V_{x}^{\mathrm{III}}{\mathrm{PO}}_{4}F$ solid-solution cathodes (*M* = Fe_1−*x*_V*_x_*; 0 < *x* < 1). But for the redox potential of V^3+/4+^ couple, the *M*–O_4_F_2_ bond length plays a dominant role in controlling the redox energy of cation V.

It is likely there is also a continuous downshift for the Fe^2+/3+^ couple shown in OCV profiles (Figure 8 and Figure 9) with increasing substitution of V^3^^+^ for Fe^3^^+^, while there is no shift for the V^3+/4+^ couple with increasing substitution of Fe^3^^+^ for V^3^^+^ in LiFe_1−*x*_V*_x_*PO_4_F (0 ≤ *x* ≤ 1), to support results of CV measurements (Figure 4). However, we do not now adopt the idea because all GITT measurements in this work started only from the fully-discharged state. It also needs to start from the fully-charged state (${\mathrm{Li}}_{1-x}{\mathrm{Fe}}_{1-x}^{\mathrm{III}}V_{x}^{\mathrm{IV}}{\mathrm{PO}}_{4}F$) at 4.5 V toward cathodic direction with identical relaxation conditions to confirm the measured OCVs including negligible kinetic effect [57]. Research is underway and will be reported elsewhere.

## 4. Materials and Methods

The VPO_4_ powder was pre-synthesized by a hydrothermal route using raw materials of H_3_PO_3_ (99% wt, Sinopharm Chem. Reag. Co. Ltd., Shanghai, China), V_2_O_5_ (99% wt, Energy Chem. Co. Ltd., Shanghai, China) and H_2_O. Firstly, H_3_PO_3_ was dissolved in H_2_O, then V_2_O_5_ was added to the solution under vigorous stirring. Reagents were placed in an autoclave, heated to 160 °C, dwelled for 6 h and cooled inside to room temperature (RT). Secondly, the intermediate product from the autoclave was dried in a vacuum oven at 80 °C for 4 h and then calcined at 800 °C for 5 h under argon in a tube furnace. The hydrothermal-synthesis process is: H_3_PO_3_ + V_2_O_5_ + H_2_O $\overset{\;160\;{}^{{}^{\circ}}C,\;6\;h\;}{\to }$ VPO_4_⋅*x*H_2_O $\overset{\;800\;{}^{{}^{\circ}}C,\;5\;h,\;\mathrm{Ar}\;}{\to }$ VPO_4_. LiFe_1−*x*_V*_x_*PO_4_F (*x* = 0, 0.1, 0.3, 0.5, 0.7, 0.9, 1) powders were then obtained by mixing the hydrothermal-synthesized VPO_4_, commercial FePO_4_ (99% wt, Mianyang Tianming New Energy Technol. Co. Ltd., Mianyang, China) and LiF (99.9% wt, Aladdin Chem. Reag. Co. Ltd., Shanghai, China), followed by pelletizing, calcining at 625 °C for 1.5 h under argon and grinding [4,26,33].

LiFe_1−*x*_V*_x_*PO_4_F (*x* = 0, 0.1, 0.3, 0.5, 0.7, 0.9, 1) electrodes and cells were prepared using the same method as pure LiFePO_4_F/LiVPO_4_F ones [4,26,33]. LiFe_1−*x*_V*_x_*PO_4_F powders, super P (conductive carbon) and binder (polyvinylidene fluoride, PVDF) were mixed to form a slurry by using *N*-methyl-pyrrolidone (NMP) as the solvent (LiFe_1−*x*_V*_x_*PO_4_F:C:PVDF = 8:1:1, % wt). The aluminum foil casted by the slurry was then vacuum-dried at 120 °C for 12 h, roller-pressed and cut into discs of 15 mm diameter (~1.767 cm^2^). The loading density of active material was 1.2–3.4 × 10^−3^ g cm^–2^ approximately. The electrolyte was 1 M LiPF_6_ dissolved in a mixture of ethylene carbonate (EC) and dimethyl carbonate (DMC) (1:1, % vol). The polypropylene film (Celgard 2400) was used as the separator and lithium foil as the counter and reference electrodes. The lithium-ion rechargeable (LIR) 2025 coin-type cells were assembled in an argon-filled glove box (Etelux Lab2000, Beijing, China).

Elaborative phase determination (8° ≤ 2θ ≤ 100°) was carried out by X-ray powder diffraction (XRD) using Cu*K*_α_ radiation (λ_α1_ = 1.54060 Å, 40 kV, 40 mA) in flat plate θ/2θ geometry at a step size of 0.01943°/step and a scan speed of 0.01203°/s (D8 Adv., Bruker Co. Ltd., Karlsruhe, Germany). Testing conditions included a divergence slit of 1.0 mm, an antiscatter slit of 6.94 mm, a primary soller slit of 2.5°, a second soller slit of 2.5° and a detector slit of 12.21 mm. Structure refinements were performed by the Rietveld method implemented in GSAS/EXIGUI Revision 1251 software [61] using the model Li_2*i*_(Fe1,V1)_1*a*_(Fe2,V2)_1*b*_{P_2*i*_[O_2*i*_]_4_}F_2*i*_ based on the LiFe_0.5_V_0.5_PO_4_F structure [25] which has only one crystallographic lithium site [7,11], contrary to the previous viewpoints [6,36,48]. Valence states of Fe/V components in FePO_4_, VPO_4_ and LiFe_1−*x*_V*_x_*PO_4_F (0 ≤ *x* ≤ 1) powders were determined by X-ray photoelectron spectra (XPS) using a Multilab 2000 spectrometer (VG Inc., Waltham, MA, USA) equipped with a focused monochromatized Al *K*_α_ X-ray source (*hν* = 1486.6 eV). All the obtained binding energy (BE) values were calibrated using the photoemission line C 1s at 284.8 eV. The microstructure and compositions of samples were characterized by a field-emission scanning electron microscope (FESEM; SU-8020, Hitachi Ltd., Tokyo, Japan) equipped with an X-ray spectrometer for energy dispersive spectroscopy (Bruker EDS QUANTAX, Karlsruhe, Germany).

To evaluate electrochemical properties of LiFe_1−*x*_V*_x_*PO_4_F (0 ≤ *x* ≤ 1) cathodes, cyclic voltammetry (CV) measurements were carried out at RT with sweep rates of 0.1/0.2/0.3/0.4/0.5 mV s^−1^, in the range of 2.0–4.5 V (vs. Li/Li^+^) for Fe^2+/3+^ and V^3+/4+^ couples, 2.0–3.6 V for Fe^2+/3+^ couple and 3.0–4.5 V for V^3+/4+^ couple, using a CHI660e electrochemical workstation (Shanghai Chenhua Instr. Co. Ltd., Shanghai, China). Galvanostatic charge/discharge tests (0.1 C) were performed at RT, in the range of 2.0−4.5 V for LiFe_1−*x*_V*_x_*PO_4_F (0 < *x* < 1), 2.0–4.0 V for LiFePO_4_F and 3.0–4.5 V for LiVPO_4_F, using a CT2001A Land battery testing system (Wuhan Land Electronics Co. Ltd., Wuhan, China). Galvanostatic intermittent titration technique (GITT) measurements started from the fully-discharged state (${\mathrm{Li}}_{2-x}{\mathrm{Fe}}_{1-x}^{\mathrm{II}}V_{x}^{\mathrm{III}}{\mathrm{PO}}_{4}F$) at 2.0 V, which realized after the cell discharged for 24 h at 0.05 C, toward anodic direction with intermittent of 5% state of charge (5% SOC, i.e., 0.05 Li^+^-extraction). The charging at 0.05 C was followed after 3 h relaxation for equilibrium at each open-circuit voltage (OCV) measuring points. GITT measurements proceeded until reaching the fully-charged state (${\mathrm{Li}}_{1-x}{\mathrm{Fe}}_{1-x}^{\mathrm{III}}V_{x}^{\mathrm{IV}}{\mathrm{PO}}_{4}F$) at 4.5 V.

## 5. Conclusions

In this work, tavorite triclinic-structured LiFe_1−*x*_V*_x_*PO_4_F (*x* = 0, 0.1, 0.3, 0.5, 0.7, 0.9 and 1) solid-solution powders, the related cathodes and Li-ion batteries were prepared and characterized.

The systematic variations in lattice parameters and unit cell volumes via XRD Rietveld refinements confirm the formation of homogeneous solid solutions, which originate from the substitution of V^3+^ for Fe^3+^ with close effective ionic radii. The valence states of Fe^3+^/V^3+^ were identified by XPS and a homogeneous distribution of Fe/V/P/F components by SEM/EDS.

A single-phase behavior is confirmed strongly by analyzing the broad shape of cyclic voltammetry (CV) peaks, sloping charge/discharge profiles and sloping open-circuit voltage (OCV) profiles in LiFe_1−*x*_V*_x_*PO_4_F solid-solution cathodes. As the vanadium content *x* increases, a downward shift in the redox potential of Fe^2+/3+^ couple was observed in CV curves. However, there was hardly any shift for the V^3+/4+^ couple. The electronegativity of *M* (*M* = Fe_1−*x*_V*_x_*) plays a dominant role compared to the *M*–O_4_F_2_ bond length for the redox potential of Fe^2+/3+^ couple. Yet for the redox potential of V^3+/4+^ couple, the *M*–O_4_F_2_ bond length plays a dominant role. The obtained diffusion coefficient of lithium ions ($D_{{\mathrm{Li}}^{+}}$) indicates that LiFe_1−*x*_V*_x_*PO_4_F (0 < *x* < 1) solid solutions have comparable electrochemical activities with their end-members (*x* = 0 or 1).

The mechanism is involved in redox energies of cations which are tuned within a wide range 2.0–4.5 V in polyanion-type cathodes, through the inductive effect introduced by cation (V for Fe) substitution.

## Supplementary Materials

The following are available online at https://www.mdpi.com/1420-3049/24/10/1893/s1, Table S1–S7: Rietveld refined parameters of the tavorite LiFe_1−_*_x_*V*_x_*PO_4_F (*x* = 0, 0.1, 0.3, 0.5, 0.7, 0.9 and 1) structure. Table S8: Comparison of lattice parameters for LiFe_1−*x*_V*_x_*PO_4_F (0 ≤ *x* ≤ 1) samples and the related publications. Figure S1: The final observed, calculated and difference profiles of the tavorite-structured LiFePO_4_F, LiFe_0.9_V_0.1_PO_4_F, LiFe_0.7_V_0.3_PO_4_F, LiFe_0.5_V_0.5_PO_4_F, LiFe_0.3_V_0.7_PO_4_F, LiFe_0.1_V_0.9_PO_4_F and LiVPO_4_F via Rietveld refinements. Figure S2: Variations of lattice parameters (***a***, ***b***, ***c***, α, β and γ) and unit cell volumes (*V*) of LiFe_1−*x*_V*_x_*PO_4_F (0 ≤ *x* ≤ 1) solid solutions. Figure S3: Scheme for a GITT measurement. Figures S4–S7: Curves of the quasi-equilibrium OCVs as a function of time by GITT, or as a function of Li^+^-extraction content *y*, plots of the slope of quasi-equilibrium OCVs as a function of Li^+^-extraction content *y* ($\delta E_{s}/\delta y$), and plots of the slope of initial transient voltage change as a function of square root of time ($\delta E/\delta t^{1/2}$), in ${\mathrm{Li}}_{1-y}{\mathrm{Fe}}_{1-x}^{\mathrm{III}}V_{x}^{\mathrm{III}}{\mathrm{PO}}_{4}F$, i.e., ${\mathrm{Li}}_{2-x-y}{\mathrm{Fe}}_{1-x}^{\mathrm{II}}V_{x}^{\mathrm{III}}{\mathrm{PO}}_{4}F$ with *x* = 0, 0.5, 0.7 and 1.

## Appendix A

Further details of crystal structures may be obtained from Cambridge Crystallographic Data Centre (CCDC)/Leibniz Institute for Information Infrastructure (FIZ Karlsruhe) joint deposition and access services (www.ccdc.cam.ac.uk; www.fiz-karlsruhe.de) on quoting the appropriate CSD numbers (G.-Q.S., J.-L.Y., S.-H.F., et al., LiFe_0.3_V_0.7_PO_4_F CSD 1906255 and LiVPO_4_F CSD 1906256, 28 March 2019).
