# Supplementary material for: Structure, Shift in Redox Potential and Li-Ion Diffusion Behavior in Tavorite LiFe1−xVxPO4F Solid-Solution Cathodes
Source: Molecules. 2019 May 16;24(10):1893. doi: 10.3390/molecules24101893 (PMC6572713; doi:10.3390/molecules24101893)
Supplement: Supplementary file 1 [file molecules-24-01893-s001.pdf]

Supplementary Materials

Structure, shift in redox potential and Li-ion diffusion behavior in tavorite $LiFe_{1-x}V_xPO_4F$ solid-solution cathodes

Jia-Li Yan, Gang-Qin Shao *, Shu-Hao Fan, Can Zhu, Yong Zhang, Jun Wang, Qi Liu

State Key Laboratory of Advanced Technology for Materials Synthesis and Processing, Wuhan University of Technology, Wuhan 430070, China; 211689@whut.edu.cn (J.-L. Yan); shuhao_fan@163.com (S.-H. Fan); zhucan@whut.edu.cn (C. Zhu); 547757900@qq.com (Y. Zhang); 941333374@qq.com (J. Wang); 593859987@qq.com (Q. Liu)

* Correspondence: gqshao@whut.edu.cn; Tel./Fax: +86-27-87879468

Table S1. Rietveld refined parameters of the tavorite $LiFePO_4F$ structure.

Type	Wyckoff	*x*	*y*	*z*	Occ.	U_{iso} (Å^2)
Li1	2*i*	0.7030(8)	0.3712(8)	0.2422(6)	1	0.0402(12)
Fe1	1*a*	0	0	0	1	0.0271(12)
Fe2	1*b*	0	0	0.5	1	0.0271(12)
P1	2*i*	0.3157(8)	0.6501(8)	0.2568(6)	1	0.0291(12)
O1	2*i*	0.3912(8)	0.2535(8)	0.5842(6)	1	0.0322(12)
O2	2*i*	0.1033(8)	-0.3364(8)	0.3750(6)	1	0.0322(12)
O3	2*i*	0.6807(8)	0.6542(8)	-0.1395(6)	1	0.0322(12)
O4	2*i*	0.2690(8)	0.7795(8)	0.1030(6)	1	0.0322(12)
F1	2*i*	0.1055(8)	0.1030(8)	0.2527(6)	1	0.0322(12)
χ^2	R_p	R_{wp}	R_{exp}	R_{F^2}		
1.18	1.44%	1.96%	1.68%	22.7%		

Space group: $P\bar{1}$ (No.2); triclinic; Z = 2; M_r = 176.76; $\rho_{cal.}$ = 3.379 g·cm^{-3}.

a = 5.1517(3) Å; b = 5.3013(2) Å; c = 7.2638(3) Å; α = 107.344(3)°; β = 108.074(3)°; γ = 98.347(3)°; V = 173.73(2) Å^3.

Table S2. Rietveld refined parameters of the tavorite $LiFe_{0.9}V_{0.1}PO_4F$ structure.

Type	Wyckoff	*x*	*y*	*z*	Occ.	U_{iso} (Å^2)
Li1	2*i*	0.290(5)	0.636(6)	0.766(5)	1	0.0141(17)
Fe1	1*a*	0	0	0	0.9	0.0011(17)
V1	1*a*	0	0	0	0.1	0.0011(17)
Fe2	1*b*	0	0	0.5	0.9	0.0011(17)
V2	1*b*	0	0	0.5	0.1	0.0011(17)
P1	2*i*	0.3182(12)	0.6554(10)	0.2564(8)	1	0.0031(17)
O1	2*i*	0.3937(12)	0.2588(10)	0.5838(8)	1	0.0061(17)
O2	2*i*	0.1058(12)	-0.3311(10)	0.3746(8)	1	0.0061(17)
O3	2*i*	0.6832(12)	0.6595(10)	-0.1399(8)	1	0.0061(17)
O4	2*i*	0.2715(12)	0.7848(10)	0.1026(8)	1	0.0061(17)
F1	2*i*	-0.1030(12)	0.1083(10)	0.2523(8)	1	0.0061(17)
χ^2	R_p	R_{wp}	R_{exp}	R_{F^2}		
1.35	1.66%	2.42%	1.80%	15.4%		

Space group: $P\bar{1}$ (No.2); triclinic; Z = 2; M_r = 176.27; $\rho_{cal.}$ = 3.378 g·cm^{-3}.

a = 5.152(2) Å; b = 5.300(2) Å; c = 7.242(3) Å; α = 107.317(6)°; β = 107.877(6)°; γ = 98.536(5)°; V = 173.3(2) Å^3.

Table S3. Rietveld refined parameters of the tavorite $LiFe_{0.7}V_{0.3}PO_4F$ structure.

Type	Wyckoff	*x*	*y*	*z*	Occ.	U_{iso} (Å²)
Li1	2*i*	0.7061(8)	0.3734(7)	0.2415(6)	1	0.0182(10)
Fe1	1*a*	0	0	0	0.7	0.0052(10)
V1	1*a*	0	0	0	0.3	0.0052(10)
Fe2	1*b*	0	0	0.5	0.7	0.0052(10)
V2	1*b*	0	0	0.5	0.3	0.0052(10)
P1	2*i*	0.3188(8)	0.6523(7)	0.2561(6)	1	0.0072(10)
O1	2*i*	0.3943(8)	0.2557(7)	0.5835(6)	1	0.0102(10)
O2	2*i*	0.1064(8)	-0.3342(7)	0.3743(6)	1	0.0102(10)
O3	2*i*	0.6838(8)	0.6564(7)	-0.1402(6)	1	0.0102(10)
O4	2*i*	0.2721(8)	0.7817(7)	0.1023(6)	1	0.0102(10)
F1	2*i*	-0.1024(8)	0.1052(7)	0.2520(6)	1	0.0102(10)
χ^2	R_p	R_{wp}	R_{exp}	R_{F^2}		
1.25	1.78%	2.52%	2.02%	11.8%		

Space group: $P\bar{1}$ (No.2); triclinic; $Z = 2$; $M_r = 175.28$; $\rho_{cal.} = 3.360$ g·cm^{-3}.

$a = 5.1532(15)$ Å; $b = 5.2994(16)$ Å; $c = 7.2401(22)$ Å; $\alpha = 107.337(4)$°; $\beta = 107.908(4)$°; $\gamma = 98.493(4)$°; $V = 173.25(14)$ Å^3.

Table S4. Rietveld refined parameters of the tavorite $LiFe_{0.5}V_{0.5}PO_4F$ structure.

Type	Wyckoff	*x*	*y*	*z*	Occ.	U_{iso} (Å²)
Li1	2*i*	0.7067(10)	0.3728(9)	0.2412(7)	1	0.0321(12)
Fe1	1*a*	0	0	0	0.5	0.0191(12)
V1	1*a*	0	0	0	0.5	0.0191(12)
Fe2	1*b*	0	0	0.5	0.5	0.0191(12)
V2	1*b*	0	0	0.5	0.5	0.0191(12)
P1	2*i*	0.3194(10)	0.6517(9)	0.2558(7)	1	0.0211(12)
O1	2*i*	0.3949(10)	0.2551(9)	0.5832(7)	1	0.0241(12)
O2	2*i*	0.1070(10)	-0.3348(9)	0.3740(7)	1	0.0241(12)
O3	2*i*	0.6844(10)	0.6558(9)	-0.1405(7)	1	0.0241(12)
O4	2*i*	0.2727(10)	0.7811(9)	0.1020(7)	1	0.0241(12)
F1	2*i*	-0.1018(10)	0.1046(9)	0.2517(7)	1	0.0241(12)
χ^2	R_p	R_{wp}	R_{exp}	R_{F^2}		
1.73	2.66%	4.15%	2.41%	21.6%		

Space group: $P\bar{1}$ (No.2); triclinic; $Z = 2$; $M_r = 174.30$; $\rho_{cal.} = 3.349$ g·cm^{-3}.

$a = 5.1556(12)$ Å; $b = 5.2991(13)$ Å; $c = 7.2138(17)$ Å; $\alpha = 107.297(4)$°; $\beta = 107.762(5)$°; $\gamma = 98.593(4)$°; $V = 172.84(11)$ Å^3.

Table S5. Rietveld refined parameters of the tavorite $LiFe_{0.3}V_{0.7}PO_4F$ structure (CSD 1906255).

Type	Wyckoff	*x*	*y*	*z*	Occ.	U_{iso} (Å²)
Li1	2*i*	0.7025(5)	0.3704(5)	0.2409(4)	1	0.0231(8)
Fe1	1*a*	0	0	0	0.3	0.0101(8)
V1	1*a*	0	0	0	0.7	0.0101(8)
Fe2	1*b*	0	0	0.5	0.3	0.0101(8)
V2	1*b*	0	0	0.5	0.7	0.0101(8)
P1	2*i*	0.3152(5)	0.6493(5)	0.2555(4)	1	0.0121(8)
O1	2*i*	0.3907(5)	0.2527(5)	0.5829(4)	1	0.0151(8)
O2	2*i*	0.1028(5)	-0.3372(5)	0.3737(4)	1	0.0151(8)
O3	2*i*	0.6802(5)	0.6534(5)	-0.1408(4)	1	0.0151(8)
O4	2*i*	0.2685(5)	0.7787(5)	0.1017(4)	1	0.0151(8)
F1	2*i*	-0.1060(5)	0.1022(5)	0.2514(4)	1	0.0151(8)
χ^2	R_p	R_{wp}	R_{exp}	R_{F^2}		
1.25	2.66%	3.61%	2.89%	9.46%		

Space group: $P\bar{1}$ (No.2); triclinic; $Z = 2$; $M_r = 173.32$; $\rho_{cal.} = 3.324$ g·cm^{-3}.

$a = 5.1642(10)$ Å; $b = 5.3022(10)$ Å; $c = 7.2052(14)$ Å; $\alpha = 107.219(3)$°; $\beta = 107.660(3)$°; $\gamma = 98.700(3)$°; $V = 173.15(9)$ Å^3.

Table S6. Rietveld refined parameters of the tavorite $LiFe_{0.1}V_{0.9}PO_4F$ structure.

Type	Wyckoff	*x*	*y*	*z*	Occ.	U_{iso} (Å²)
Li1	2*i*	0.7077(18)	0.3743(18)	0.2368(13)	1	0.015(2)
Fe1	1*a*	0	0	0	0.1	0.002(2)
V1	1*a*	0	0	0	0.9	0.002(2)
Fe2	1*b*	0	0	0.5	0.1	0.002(2)
V2	1*b*	0	0	0.5	0.9	0.002(2)
P1	2*i*	0.3204(18)	0.6532(18)	0.2514(13)	1	0.004(2)
O1	2*i*	0.3959(18)	0.2566(18)	0.5788(13)	1	0.007(2)
O2	2*i*	0.1080(18)	-0.3333(18)	0.3696(13)	1	0.007(2)
O3	2*i*	0.6854(18)	0.6573(18)	-0.1449(13)	1	0.007(2)
O4	2*i*	0.2737(18)	0.7826(18)	0.0976(13)	1	0.007(2)
F1	2*i*	-0.1008(18)	0.1061(18)	0.2473(13)	1	0.007(2)
χ^2	R_p	R_{wp}	R_{exp}	R_{F^2}		
3.48	7.08%	12.6%	3.62%	20.0%		

Space group: $P\bar{1}$ (No.2); triclinic; $Z = 2$; $M_r = 172.34$; $\rho_{cal.} = 3.303$ g·cm^{-3}.
$a = 5.1643(6)$ Å; $b = 5.2953(8)$ Å; $c = 7.2260(8)$ Å; $\alpha = 107.313(13)°$; $\beta = 107.687(15)°$; $\gamma = 98.665(12)°$; $V = 173.31(2)$ Å³.

Table S7. Rietveld refined parameters of the tavorite $LiVPO_4F$ structure (CSD 1906256).

Type	Wyckoff	*x*	*y*	*z*	Occ.	U_{iso} (Å²)
Li1	2*i*	0.7030(6)	0.3689(6)	0.2402(5)	1	0.0250(9)
V1	1*a*	0	0	0	1	0.0120(9)
V2	1*b*	0	0	0.5	1	0.0120(9)
P1	2*i*	0.3157(6)	0.6478(6)	0.2548(5)	1	0.0140(9)
O1	2*i*	0.3912(6)	0.2512(6)	0.5822(5)	1	0.0170(9)
O2	2*i*	0.1033(6)	-0.3387(6)	0.3730(5)	1	0.0170(9)
O3	2*i*	0.6807(6)	0.6519(6)	-0.1415(5)	1	0.0170(9)
O4	2*i*	0.2690(6)	0.7772(6)	0.1010(5)	1	0.0170(9)
F1	2*i*	-0.1055(6)	0.1007(6)	0.2507(5)	1	0.0170(9)
χ^2	R_p	R_{wp}	R_{exp}	R_{F^2}		
1.79	5.50%	7.73%	4.32%	12.0%		

Space group: $P\bar{1}$ (No.2); triclinic; $Z = 2$; $M_r = 171.85$; $\rho_{cal.} = 3.274$ g·cm^{-3}.
$a = 5.1770(14)$ Å; $b = 5.3096(16)$ Å; $c = 7.2353(21)$ Å; $\alpha = 107.412(4)°$; $\beta = 107.748(5)°$; $\gamma = 98.565(4)°$; $V = 174.31(13)$ Å³.

Table S8. Comparison of lattice parameters for $LiFe_{1-x}V_xPO_4F$ ($0 \le x \le 1$) samples and the related publications.

Materials	*a* (Å)	*b* (Å)	*c* (Å)	α (°)	β (°)	γ (°)	*V* (Å³)	Remark
$LiFePO_4F$ [1]	5.1551(3)	5.3044(3)	7.2612(4)	107.356(5)	107.855(6)	98.618(5)	173.91(2)	–
$LiFePO_4F$ [2]	5.3002(2)	7.2601(2)	5.1516(2)	107.880(3)	98.559(3)	107.343(3)	173.67(6)	–
*$LiFePO_4F$ (ICSD 428404, ICDD-I04003) [3]	5.1516(2)	5.2985(2)	7.2580(2)	107.316(4)	107.952(3)	98.493(3)	173.558(6)	*our previous work
$LiFePO_4F$	5.1517(3)	5.3013(2)	7.2638(3)	107.344(3)	108.074(3)	98.347(3)	173.73(2)	
$LiFe_{0.9}V_{0.1}PO_4F$	5.152(2)	5.300(2)	7.242(3)	107.317(6)	107.877(6)	98.536(5)	173.3(2)	
$LiFe_{0.7}V_{0.3}PO_4F$	5.1532(15)	5.2994(16)	7.2401(22)	107.337(4)	107.908(4)	98.493(4)	173.25(14)	
$LiFe_{0.5}V_{0.5}PO_4F$	5.1556(12)	5.2991(13)	7.2138(17)	107.297(4)	107.762(5)	98.593(4)	172.84(11)	this work
$LiFe_{0.3}V_{0.7}PO_4F$ (CSD 1906255)	5.1642(10)	5.3022(10)	7.2052(14)	107.219(3)	107.660(3)	98.700(3)	173.15(9)	
$LiFe_{0.1}V_{0.9}PO_4F$	5.1643(6)	5.2953(8)	7.2260(8)	107.313(13)	107.687(15)	98.665(12)	173.31(2)	
$LiVPO_4F$ (CSD 1906256)	5.1770(14)	5.3096(16)	7.2353(21)	107.412(4)	107.748(5)	98.565(4)	174.31(13)	
$LiVPO_4F$ (ICSD 184601) [4]	5.1708(3)	5.3083(3)	7.2631(4)	107.595(3)	107.969(2)	98.388(2)	174.36(2)	–
$LiVPO_4F$ (ICSD 183876) [5]	5.3094(1)	7.4993(6)	5.1688(8)	112.933(0)	81.664(0)	113.125(0)	174.31	–
$LiVPO_4F$ [6]	5.1684(2)	5.3080(2)	7.2635(3)	107.563(3)	108.086(3)	98.294(2)	174.25(1)	–
*$LiVPO_4F$ [7]	5.1704(4)	5.3057(5)	7.2545(6)	107.480(8)	107.981(8)	98.410(7)	174.167(16)	*our previous work
$LiFe_{0.5}V_{0.5}PO_4F$ [8]	5.1573(1)	5.2978(2)	7.2409(2)	107.424(3)	107.945(2)	98.431(2)	173.25(1)	

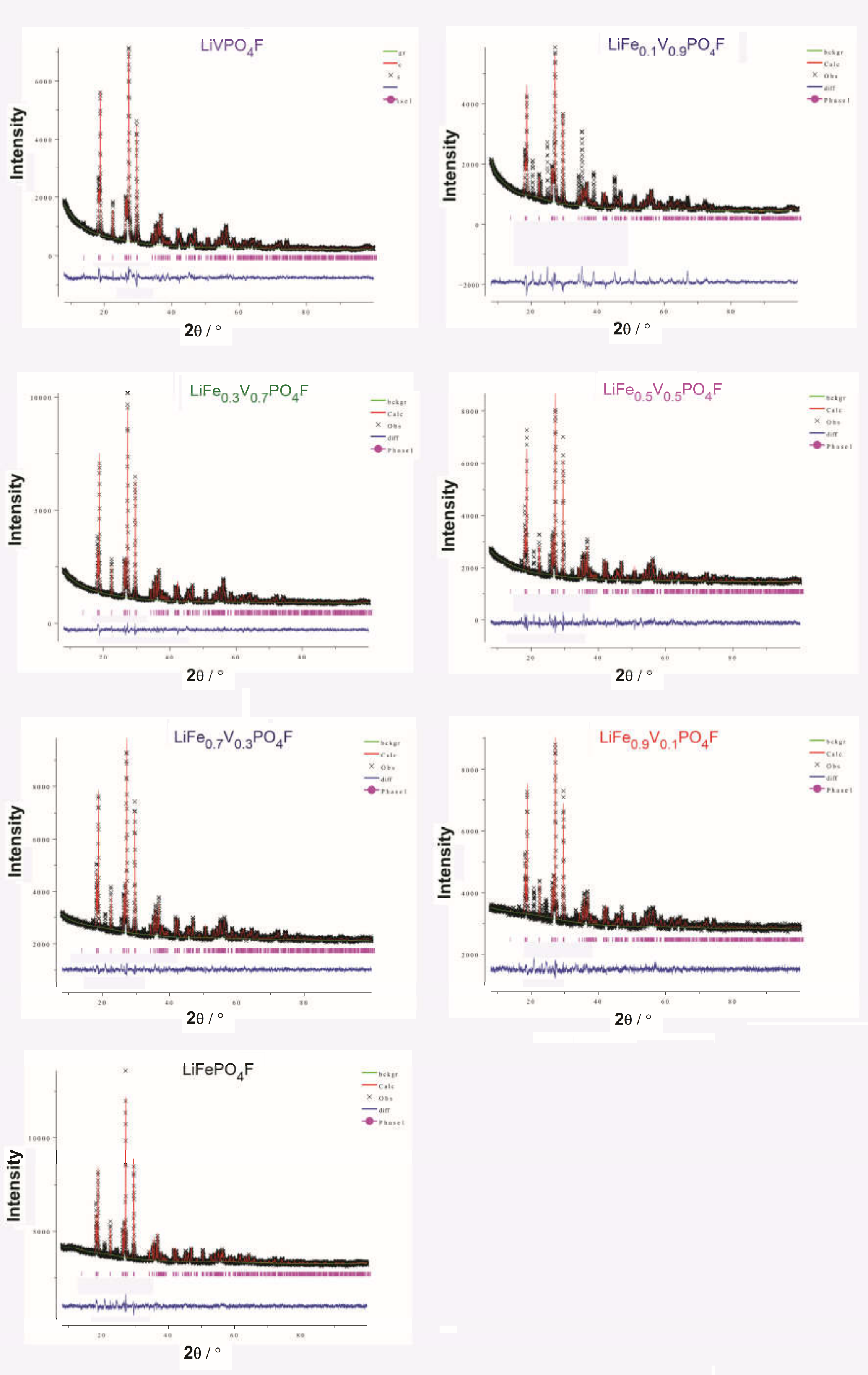

Figure S1. The final observed, calculated and difference profiles of the tavorite-structured $LiFePO_4F$ (**a**), $LiFe_{0.9}V_{0.1}PO_4F$ (**b**), $LiFe_{0.7}V_{0.3}PO_4F$ (**c**), $LiFe_{0.5}V_{0.5}PO_4F$ (**d**), $LiFe_{0.3}V_{0.7}PO_4F$ (**e**), $LiFe_{0.1}V_{0.9}PO_4F$ (**f**) and $LiVPO_4F$ (**g**) *via* Rietveld refinements.

Figure S2 shows variations of lattice parameters (***a***, ***b***, ***c***, α, β & γ) and unit cell volumes (V) of $LiFe_{1-x}V_xPO_4F$ ($0 \le x \le 1$) solid solutions. The volume deviation (ΔV) between $LiFePO_4F$ and $LiVPO_4F$ is under 0.4%, much less

than that between $LiFePO_4F$ and $LiVPO_4O$ (1.3%). In this work, the *V* values of the prepared $LiFePO_4F$–$LiVPO_4F$ samples are located in a narrow region due to the close effective ionic radii of Fe^{3+} (0.645 Å) and V^{3+} (0.640 Å), indicating the formation of solid solutions.

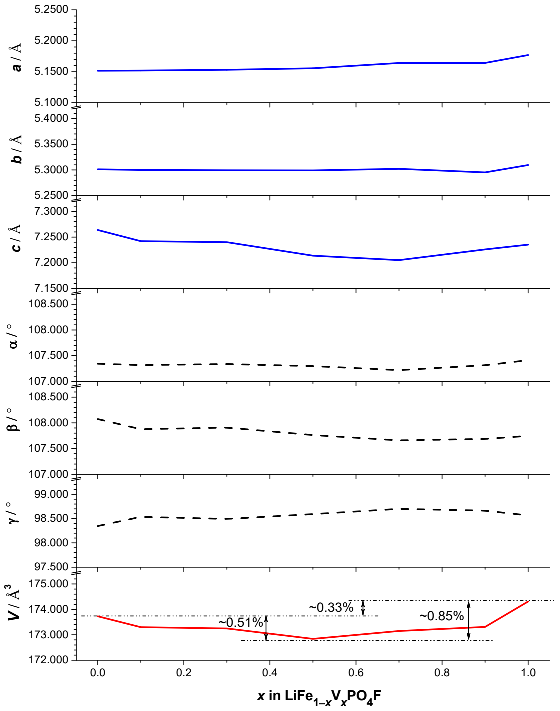

Figure S2. Variations of lattice parameters (***a, b, c,*** α, β & γ) and unit cell volumes (*V*) of $LiFe_{1-x}V_xPO_4F$ ($0 \le x \le 1$) solid solutions.

Figure S3 shows a scheme for the GITT measurement [9]. The change rate of the steady-state voltage ($\delta E_s/\delta t$) during a single-step GITT measurement is 6.7 μV s^{-1}.

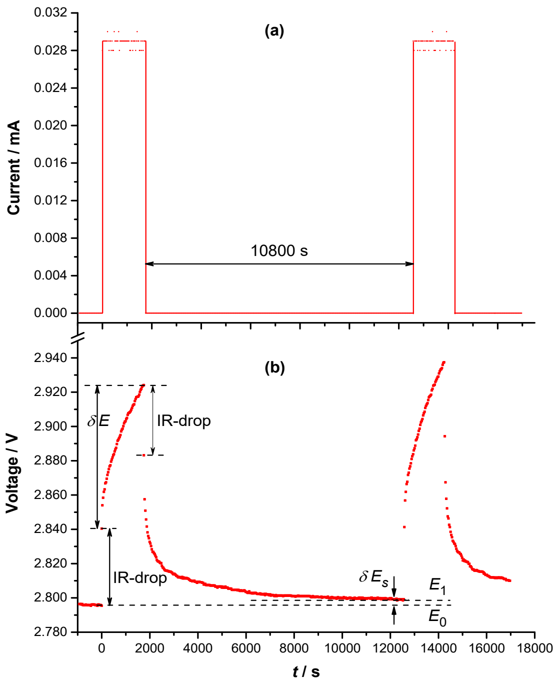

Figure S3. Scheme for a GITT measurement. (**a**) the constant current pulse; (**b**) potential response.
E_0——steady-state potential prior to the constant current pulse (V); E_1——steady-state potential after the constant current pulse (V); I——constant current pulse (A); R——internal resistance (Ω); δE——total change of cell voltage during a constant current pulse of a single-step GITT measurement neglecting the IR-drop (V); δE_s——change of the steady-state voltage during a single-step GITT measurement (V).

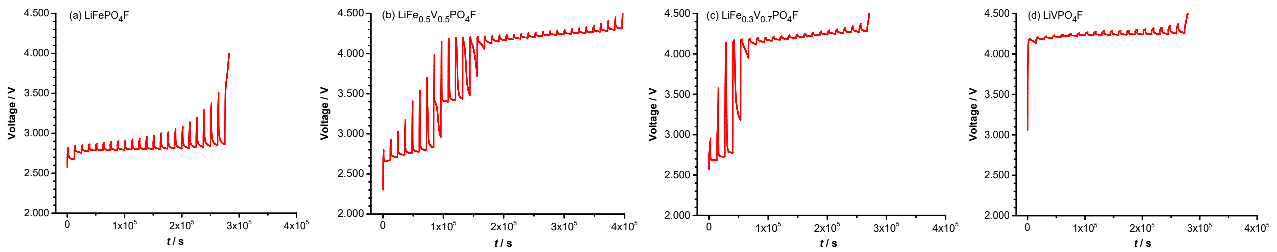

Figure S4. Curves of the quasi-equilibrium OCVs as a function of time by GITT in $Li_{1-y}Fe^{III}_{1-x}V^{III}_{x}PO_4F$, *i.e.* $Li_{2-x-y}Fe^{II}_{1-x}V^{III}_{x}PO_4F$ with x = 0 (**a**), x = 0.5 (**b**), x = 0.7 (**c**) and x = 1 (**d**).

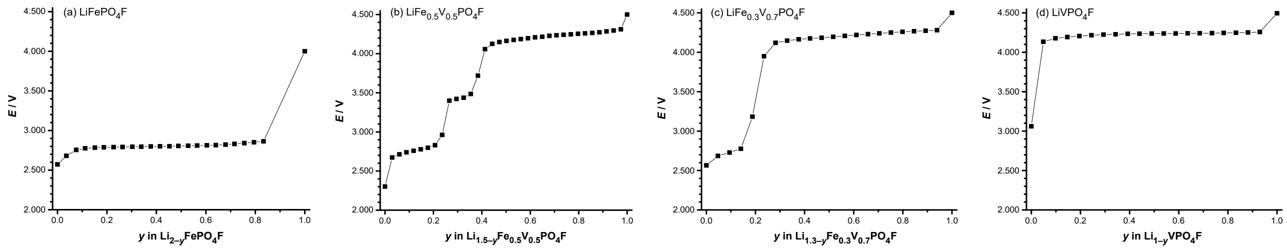

Figure S5. Curves of the quasi-equilibrium OCVs as a function of Li⁺-extraction content y by GITT in $Li_{1-y}Fe^{III}_{1-x}V^{III}_{x}PO_4F$, *i.e.* $Li_{2-x-y}Fe^{II}_{1-x}V^{III}_{x}PO_4F$ with x = 0 (**a**), x = 0.5 (**b**), x = 0.7 (**c**) and x = 1 (**d**).

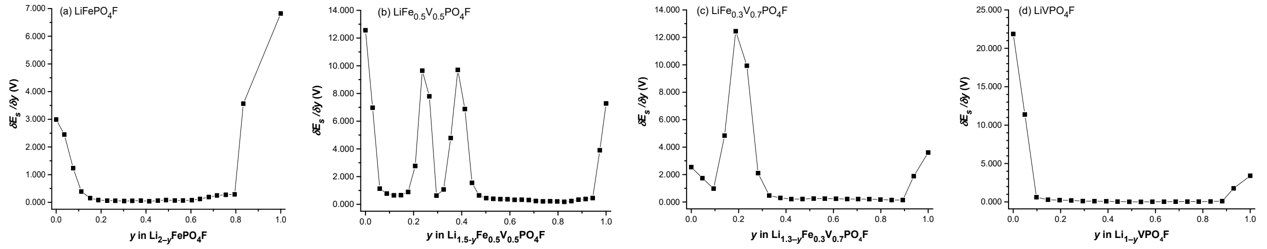

Figure S6. Plots of the slope of quasi-equilibrium OCVs as a function of Li⁺-extraction content y ($\delta E_s/\delta y$) and the fitted lines in $Li_{1-y}Fe^{III}_{1-x}V^{III}_{x}PO_4F$, *i.e.* $Li_{2-x-y}Fe^{II}_{1-x}V^{III}_{x}PO_4F$ with x = 0 (**a**), x = 0.5 (**b**), x = 0.7 (**c**) and x = 1 (**d**).

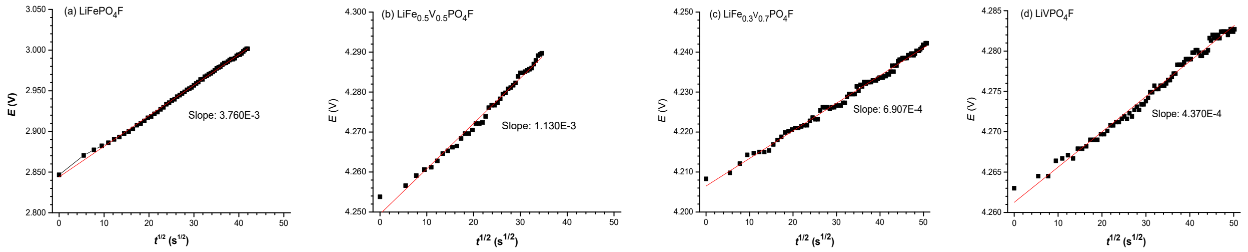

Figure S7. Plots of the slope of initial transient voltage change as a function of square root of time ($\delta E/\delta t^{1/2}$, within a single charging current pulse duration) and the fitted lines in $Li_{1-y}Fe^{III}_{1-x}V^{III}_{x}PO_4F$, *i.e.* $Li_{2-x-y}Fe^{II}_{1-x}V^{III}_{x}PO_4F$ with x = 0 (**a**), x = 0.5 (**b**), x = 0.7 (**c**) and x = 1 (**d**).

References

1. Recham, N.; Chotard, J.N.; Jumas, J.C.; Laffont, L.; Armand, M.; Tarascon, J.M. Ionothermal synthesis of Li-based fluorophosphates electrodes. *Chem. Mater.* **2010**, *22*, 1142–1148; DOI: 10.1021/cm9021497.
2. Ramesh, T.N.; Lee, K.T.; Ellis, B.L.; Nazar, L.F. Tavorite lithium iron fluorophosphate cathode materials phase transition and electrochemistry of $LiFePO_4F$-Li_2FePO_4F. *Electrochem. Solid-State Lett.* **2010**, *13*, A43–A47; DOI: 10.1149/1.3298353.
3. Chen, D.; Shao, G.-Q.; Li, B.; Zhao, G.-G.; Li, J.; Liu, J.-H.; Gao, Z.-S.; Zhang, H.-F. Synthesis, crystal structure and electrochemical properties of $LiFePO_4F$ cathode material for Li-ion batteries. *Electrochim. Acta* **2014**, *147*, 663–668; DOI: 10.1016/j.electacta.2014.09.131.
4. Mba, J.M.A.; Masquelier, C.; Suard, E.; Croguennec, L. Synthesis and crystallographic study of homeotypic $LiVPO_4F$ and $LiVPO_4O$. *Chem. Mater.* **2012**, *24*, 1223–1234; DOI: 10.1021/cm3003996.
5. Ellis, B.L.; Ramesh, T.N.; Davis, L.J.M.; Goward, G.R.; Nazar, L.F. Structure and electrochemistry of two-electron redox couples in lithium metal fluorophosphates based on the tavorite structure. *Chem. Mater.* **2011**, *23*, 5138–5148; DOI: 10.1021/cm201773n.
6. Onoda, M.; Ishibashi, T. Phase transition and spin dynamics of the $LiVPO_4F$ insertion electrode with the S = 1 linear chain and the development of F–O mixed system. *J. Phys. Soc. Jpn.* **2015**, *84*, 044802(1–5); DOI: 10.7566/jpsj.84.044802.
7. Li, B. Preparation and electrochemical properties of $LiVPO_4F$ cathode material for Li-ion batteries. Master Thesis, Wuhan University of Technology, Wuhan, Hubei, China, 2012.
8. Huang, Z.-D.; Orikasa, Y.; Masese, T.; Yamamoto, K.; Mori, T.; Minato, T.; Uchimoto, Y. A novel cationic-ordering fluoro-polyanionic cathode $LiV_{0.5}Fe_{0.5}PO_4F$ and its single phase Li^+ insertion/extraction behaviour. *RSC Adv.* **2013**, *3*, 22935–22939; DOI: 10.1039/c3ra44094j.
9. Deiss, E. Spurious chemical diffusion coefficients of Li in electrode materials evaluated with GITT. *Electrochim. Acta* **2005**, *50*, 2927–2932; DOI: 10.1016/j.electacta.2004.11.042.

1. Recham, N.; Chotard, J.N.; Jumas, J.C.; Laffont, L.; Armand, M.; Tarascon, J.M. Ionothermal Synthesis of Li-Based Fluorophosphates Electrodes†. *Chemistry of Materials* **2010**, *22*, 1142-1148, doi:10.1021/cm9021497.
2. Ramesh, T.N.; Lee, K.T.; Ellis, B.L.; Nazar, L.F. Tavorite Lithium Iron Fluorophosphate Cathode Materials Phase Transition and Electrochemistry of LiFePO4F-Li2FePO4Fpdf. *Electrochemical and Solid-State Letters* **2010**, *13*, A43-A47.
3. Chen, D.; Shao, G.Q.; Li, B.; Zhao, G.G.; Li, J.; Liu, J.H.; Gao, Z.S.; Zhang, H.F. Synthesis, crystal structure and electrochemical properties of LiFePO4F cathode material for Li-ion batteries. *Electrochimica Acta* **2014**, *147*, 663-668, doi:10.1016/j.electacta.2014.09.131.
4. Mba, J.-M.A.; Masquelier, C.; Suard, E.; Croguennec, L. Synthesis and Crystallographic Study of Homeotypic LiVPO4F and LiVPO4O. *Chemistry of Materials* **2012**, *24*, 1223-1234, doi:10.1021/cm3003996.
5. Ellis, B.L.; Ramesh, T.N.; Davis, L.J.M.; Goward, G.R.; Nazar, L.F. Structure and Electrochemistry of Two-Electron Redox Couples in Lithium Metal Fluorophosphates Based on the Tavorite Structure. *Chemistry of Materials* **2011**, *23*, 5138-5148, doi:10.1021/cm201773n.
6. Onoda, M.; Ishibashi, T. Phase Transition and Spin Dynamics of the LiVFPO4 Insertion Electrode with the S= 1 Linear Chain and the Development of F–O Mixed System. *J. Phys. Soc. Jpn.* **2015**, *84*, 044802.
7. Li, B. Preparation and Electrochemical Properties of $LiVPO_4F$ Cathode Material for Li-ion Batteries. *Master Thesis, Wuhan University of Technology, Wuhan, Hubei, China* **2012**.
8. Huang, Z.D.; Orikasa, Y.; Masese, T.; Yamamoto, K.; Mori, T.; Minato, T.; Uchimoto, Y. A novel cationic-ordering fluoro-polyanionic cathode LiV0.5Fe0.5PO4F and its single phase Li+ insertion/extraction behaviour. *Rsc Advances* **2013**, *3*, 22935-22939.
9. Deiss, E. Spurious chemical diffusion coefficients of Li in electrode materials evaluated with GITT. *Electrochimica Acta* **2005**, *50*, 2927-2932.
